# Assessment of sponge sampling for real-time PCR detection of *Cystoisospora suis* from environmental and faecal samples from piglet-producing farms

**DOI:** 10.1186/s40813-025-00454-5

**Published:** 2025-07-31

**Authors:** Hendrik Loesing, Stefanie Bartelt, Vojislav Cvjetkovic, Christina Soeckler-Lionetti, Larissa Bechmann, Kerstin Kipschull, Thomas Blondel, James Mills, Nicolas Guerra, Daniel Sperling

**Affiliations:** 1 vetxpertsvetxperts GmbH, Carl-Benz-Straße 21, 48734 Reken, Germany; 2 Ceva Tiergesundheit GmbH, Kanzlerstrasse 4, 40472 Düsseldorf, Germany; 3 Bio-Diagnostix Labor GmbH, Carl-Benz-Straße 21, 48734 Reken, Germany; 4 Ceva Santé Animale, 10 Avenue de La Ballastière, 33500 Libourne, France

**Keywords:** *Cystoisospora suis*, Real-time PCR, Sponge sampling, Faecal sampling, Cystoisosporosis

## Abstract

**Background:**

*Cystoisospora suis* (*C. suis*) infects piglets in their first week of life and can subsequently lead to diarrhoea and production losses. The detection of *C. suis* oocysts relies mostly on the collection of piglet faeces as sampling material and analysis through flotation and autofluorescence microscopy, which involves repeated sampling. The objective of the present study was to evaluate the use of sponges for environmental sampling for the detection of *C. suis* via real-time PCR and its suitability for surveillance programs applied on farms.

**Results:**

All farms included in the study were positive for *C. suis* according to qPCR, with positivity rates ranging from 20 to 100%. The frequency of positive cases was 68% for faecal samples and 67% for samples collected by sponges. The alignment between the different sampling protocols was 100% achieved at the farm level. In the case of the individual pair samples, a difference in 27 samples was observed (10.8%). Considering the faecal sampling strategy as the gold standard, the sensitivity of the sampling protocol with sponges was 91.2%, and the specificity was 84.8%. Compared with the sponge-collected samples, the faecal samples presented slightly greater DNA masses, with a correlation coefficient of r = 0.51, indicating a moderate positive relationship between the two sampling methods. An influence towards a higher DNA load with samples with a pasty and semiliquid consistency was observed, mainly in the case of the sponge technique.

**Conclusions:**

Our results indicate that the collection of faecal samples at the litter or farm level and comparisons with environmental samples yield similar detection rates when sampling is combined with qPCR. From that perspective, the use of sponges for the detection of *C. suis* in organic material from the farrowing crate environment can be considered a good alternative to the more laborious and time-consuming collection of faecal samples.

**Supplementary Information:**

The online version contains supplementary material available at 10.1186/s40813-025-00454-5.

## Background

The protozoan parasite *Cystoisospora suis* (*C. suis*) usually infects piglets in their first week of life and can subsequently lead to diarrhoea. The severity of infection depends on several factors, including age and coinfections [1]. After oral ingestion of sporulated oocysts and a latency period of approximately five days, *C. suis* replicates in the small intestine. This can lead to villus atrophy as well as erosion of the mucosal tissue [1, 2]. Whereas morbidity is usually high and mortality rather low, mortality has been found to be up to 38% higher in early-infected, nontreated piglets with an additional *Clostridium perfringens* type A coinfection [3, 4]. However, in *C. suis* infections, clinical signs might not be present at all, and pigs sometimes show only reduced biological performance [5]. Subclinical infections with *C. suis* have been reported to have a negative effect on average daily gain in suckling piglets, with a total reduction of up to 1000 g of live weight at weaning [5, 6]. On farms where piglets test positive for *C. suis*, toltrazuril (TZL) is the preferred substance for the treatment of the parasite, as other effective treatment options are currently not available [7].

Oocysts, the final stages of development, are not excreted at a constant rate. The shedding pattern is bimodal, with the first detectable excretion peak occurring five to six days after infection. Oocyst counts decrease sharply between 7 and 9 days postinfection, followed by a second smaller peak between 11 and 13 days postinfection [1, 8].

Addressing *C. suis* infection relies on laboratory diagnostics, metaphylactic treatment, and adequate cleaning and disinfection protocols [1, 9].

Although sulfonamides were first reported to be effective against *C. suis*, toltrazuril has been shown to be more efficient and practical in real-life use [10, 11]. Metaphylactic treatment is justified since clinical symptoms appear up to one week after infection, and infected piglets have high excretion rates [12]. Despite the high efficacy of TZL, evidence of resistance has been recorded and described in the field [13]. The crucial aspect in addressing *C. suis* is adequate cleaning and disinfection as part of the control program applied on positive farms. It is important to mechanically clean the farrowing pens after weaning and use appropriate detergents to remove grease from rough surfaces. The disinfectant should be able to destroy the oocysts and should be based on oxygen releasers and/or chlorocresol-containing compounds [14]. Autofluorescence microscopy of faecal smears is the preferred method for determining the presence of oocysts and infection on a farm; however, it requires several sampling time points to cover previously described shedding peaks [8, 9]. Furthermore, not all laboratories are standardly equipped with such microscopes, and their staff are trained to use them [8]. Molecular diagnostic techniques for the detection of *C. suis* have been described since the beginning of this century [15–18]. Recently, real-time PCR method that relies on suckling piglet faeces has been developed and can be used in commercial laboratories [19].

The detection of *C. suis* oocysts relies mostly on suckling piglet faeces collected as sampling material. The oocysts can be analysed through flotation with McMaster, which provides a quantitative assessment, and a qualitative-based method, autofluorescence microscopy, both of which implicate repeated sampling at the 2nd and 3rd weeks of age [8, 9]. A previous study evaluated the PCR technique and found that it exhibited higher analytical sensitivity than autofluorescence and that McMaster counting once is sufficient to achieve high sensitivity; however, it is still necessary to enter the farrowing pens and fill the collection tubes with approximately the same amount of faecal material [20]. Compared with techniques based on the visualization of oocysts, PCR-based techniques may detect small amounts of parasite DNA in shed cells, with faeces even in the pre- or postpatent periods, providing a method for early detection [20]. Manual collection of faeces can be time-consuming and even dangerous, especially in the case of aggressive sows and free farrowing, a system that has recently grown in popularity.

Experiences from veterinary bacteriology have shown that environmental sampling and related programs can be considered integral parts of a routine surveillance protocol, such as for *Salmonella* spp. and Shiga toxin*-*producing *Escherichia coli* monitoring [21, 22]. Such sampling approach, which is based on environmental sampling, can be used for early detection of contamination and identification of farms at risk and as components of infection management programs. The sponge sampling of environmental and faecal samples can be easily performed without needing to enter pens and disturb the animals [23]. Furthermore, it is less stressful for piglets and sows. Given this context, the goal of this study was to evaluate the use of sponges for the collection of environmental samples and compare this method with the collection of faecal material for the detection of *C. suis* via a sampling technique with consequent real-time PCR detection. The sponge collection of environmental samples may serve as an alternative sampling strategy to the current procedures applied in the diagnostics of *C. suis*. We hypothesize that a sponge sampling strategy offers similar sensitivity to classical sampling approaches (faecal collection) to detect *C. suis* on farms and at the litter level.

## Methods

For the present study, 25 piglet-producing farms with an average of 617 (± 412) sows were selected according to their history of *C. suis* infection and their willingness to participate. Attending veterinarians at the sites were responsible for sampling faecal and environmental material as part of a standard diagnostic service provided. Most types of sites were farrow-to-finish farms (n = 12; 48%), followed by producers of grower pigs weighing 25 kg (n = 11; 44%), one baby piglet and one gilt. Farmers or animal owners signed a consent form prior to participating in both studies (Supplementary Material 1). The age of the piglets at the time of sampling was between 2 and 3 weeks.

**Table:** 

Farm	Production type	Herd size (sows)	Number of workers	Uses Toltrazuril	Toltrazuril until 3rd day	Neonatal diarrhea history	Daily cleaning farrowing	All in/ All out farrowing	Cleaning, disinfection, both	Anticoccidial disinfectant
1	PP	1100	6–10	Yes	Yes	No	Yes	Yes	Both	N/A
2	PN	1600	6–10	No	N/A	Yes	Yes	No	Both	No
3	PN	510	3–5	Yes	Yes	Yes	Yes	Yes	Both	No
4	PN	200	1–2	No	N/A	Yes	Yes	Yes	Cleaning	N/A
5	PN	600	3–5	Yes	Yes	No	Yes	No	Both	No
6	PN	520	1–2	Yes	Yes	No	Yes	Yes	Both	N/A
7	PN	320	1–2	No	N/A	No	Yes	Yes	Both	Yes
8	PN	650	3–5	No	N/A	No	Yes	Yes	Both	Yes
9	PN	650	3–5	No	N/A	Yes	No	Yes	Both	Yes
10	PN	240	1–2	No	N/A	No	Yes	Yes	Both	Yes
11	PN	385	1–2	No	N/A	Yes	Yes	Yes	Both	Yes
12	PN	1800	6–10	No	N/A	Yes	Yes	Yes	Both	Yes
13	FF	650	1–2	No	N/A	Yes	No	Yes	Both	Yes
14	FF	700	3–5	No	N/A	No	N/A	Yes	Both	Yes
15	FF	330	1–2	Yes	No	Yes	No	Yes	Both	Yes
16	FF	900	6–10	No	N/A	No	N/A	N/A	N/A	No
17	FF	250	1–2	No	N/A	No	Yes	Yes	Both	Yes
18	GP	120	1–2	No	N/A	No	No	Yes	Both	Yes
19	PC	950	6–10	No	N/A	Yes	Yes	Yes	Both	Yes
20	PC	1000	1–2	Yes	Yes	Yes	Yes	Yes	Cleaning	N/A
21	PC	335	3–5	No	N/A	No	Yes	Yes	Both	No
22	PC	330	1–2	No	N/A	Yes	Yes	Yes	Both	N/A
23	PC	240	1–2	No	N/A	Yes	Yes	Yes	Both	Yes
24	PC	390	3–5	Yes	Yes	No	Yes	Yes	Both	Yes
25	PC	650	3–5	No	N/A	No	No	Yes	Both	Yes

FF: Farrow-to-finish farm, PP: Piglet production farm, FN: Farrowing and nursery farm, GP: Gilt production farm, PC: Partly closed farm; N/A: No answer provided

The investigator was provided with a ready-to-use cardboard kit containing the following elements: a short summary of sampling instructions (Supplementary Material 2), one pair of latex gloves (Franz Mensch, Buchloe, Germany), an investigation form (Supplementary Material 3), ten labelled (1a–1e; 2a–2e) faecal collection tubes (12 ml) with a sampling spoon (FloraCura GmbH, Garmisch-Partenkirchen, Germany), ten scouring sponges (10.5 cm × 7.5 cm; Jantex, Bristol, United Kingdom), 12 labelled (1–10) plastic bags (120 mm × 170 mm × 60 µm; Welter Services Einzelunternehmen, Neunkirchen-Seelscheid, Germany), a ballpoint pen (Hauptfleisch, Karlsruhe, Germany) and a foam-based cool pack (114 × 102 × 19 mm; VWR International, Ismaning, Germany).

In total, ten farrowing crates were sampled per farm [9]. From each farrowing crate, the investigator collected five individual fresh faecal samples from different locations on the ground and pooled them into a collection tube, ideally filling approximately 75% (9 ml) of its total volume. At the same time, the scouring sponge was used to collect samples from three areas of the environment in which piglets were kept: (i) first, the walls of the farrowing crate were wiped, then (ii) the floor was wiped, and (iii) the faecal material that was wiped from these spots was collected. Sponge collection of faecal material occurred after the collection of faecal samples via wiping. Finally, every sponge was placed into the corresponding plastic bag and sealed. Sampling and shipping were performed two days later at the latest.

Upon delivery to the laboratory (Bio-Diagnostix Labor GmbH, Reken, Germany), the consistency of the faecal samples within the collection tubes was evaluated according to the following categories: 1 = firm, 2 = pasty, 3 = half-liquid and 4 = liquid. [9]. To wash the organic material from the sponges, buffered peptone water (4 ml) was added to the sponges in the plastic bags. The bags were then sealed and kneaded so that the faecal material could dissolve. Two millilitres of dissolved faeces were transferred into centrifuge tubes and centrifuged at a relative centrifugal force of r = 2147 for three minutes. The supernatant was discarded, and the pellet was stored.

For DNA extraction via the QIAamp Power Fecal Pro DNA Kit (Qiagen), 0.1 g of the faecal samples or the pellets were used. The kit was run according to the manufacturer’s protocols with the following modifications: Instead of 0.25 g of the faecal sample or pellet material, only 0.1 g were used, on the basis of previous adoption and implementation of the method. The samples were then homogenized in tubes filled with garnet shards in a Bioprep-6 homogenizer and vortexed for 20 min at 2500 rpm. In addition, when the CD3 solution was added, 0.4 μl of Xeno-DNA was added as an extraction control.All centrifugation steps were performed at 15,000 r.

A qPCR assay was performed according to the previously used protocol with primers specific for the ITS1 region of the *C. suis* genome [19, 24]. In brief, the qPCRs were run with a QuantStudio 5 qPCR machine (Biosystems) using a QuantiNova Probe RT‒PCR Kit (Qiagen). Reactions were performed in a final volume of 20 μl with 4 μl of DNA sample, 2.4 μl of nuclease-free water, 10 μl of QuantiNova MasterMix (2X), 0.1 μl of ROX reference dye, 1 μl each of forwards and reverse primers (10 μM): CystoisoITSGF1 (GATCATTCACACGTGGCCCTT) and CystoisoITSR4 (CCTTTATGCGGCCATTCGAAGC), 0.5 μl of probe (10 μM) and 1 µl of BIPC-prime-time-Assay, with product target size 147 bp. The running conditions were as follows: initial denaturation at 95 °C for 10 min, followed by 40 cycles of 95 °C for 10 s and 60 °C for 40 s.

Additionally, a positive control (gBlock) containing the *C. suis* ITS region (TAGTAATGCAGACACTTGCGGTCCATCTCGGATCATTCACACGTGGCCCTTGAAACATCTTTCAAACCTTTAGCAACTGAATCCCCATATTGATGGTCATAAGGAACAGCCTGCTGAGCGTTCGGGGGAAGCTGTGTTTCCCCGCGCCGCCGGCTGCTTC.

GAATGGCCGCATAAAGGTGCATGATCTACGTGCGTCACATGCAGTAC) and a negative template control—nuclease-free water—were tested on the same plate.

The protocol is described in the patent application WO 2022/074059 A1, entitled Method for detecting *Cytoisospora suis*.

### Statistical analysis

Numerical data (CT values) are summarized as means with standard deviations, medians, first quartiles, third quartiles, minimums and maxima.

Categorical data (Faecal score) were summarized using the number of observations and percentages.

To evaluate the agreement of detection of *C suis* between sponges and faecal analysis, a Bland‒Altman analysis was performed. This method allows us to evaluate the bias between the mean differences of the two methods.

For faecal score comparison, a linear regression model was used with CT values as the response variable and the faecal scores as a covariate. Least square means with 95% confidence intervals and p values were calculated. The assumptions of normality, homoscedasticity and linearity were checked.

The sensitivity, specificity, and positive and negative predictive values were also calculated, with CT values from sponges used as references.

R software version 4.2.2 was used for the statistical analyses [25].

## Results

All farms included in the study were positive for *C. suis* according to qPCR, with rates ranging from 20 to 100%, irrespective of the sampling method. The most frequently observed faecal score from the samples examined was FS 2 (pasty consistency), representing 67.6% of the samples, followed by firm faeces (FS1) (24.4%) and semiliquid samples (FS3) (8.0%). No liquid faecal consistency (FS4) was recorded in the submitted samples.

The average age of the piglets was 17.73 (standard deviation ± 3.94) days.

The frequency of positive cases (500 samples) was 68% for faecal samples and 67% for samples collected by sponges. The alignment between the different sampling protocols was 100% at the farm level. In the case of the individual sample pairs, differences were observed in 27 samples (10.8%).

Considering the collection of faecal samples as the established sampling strategy, the sensitivity of the sponge sampling protocol was 91.2%, and the specificity was 84.8% (Table 1).

**Table 1:** Frequencies of positive and negative samples with faecal samples considered the standard

Levels	Positive faeces, n (%)	Negative faeces, n (%)
Positive	156 (91.23)	12 (15.19)
Negative	15 (8.77)	67 (84.81)

**Fig. 1:**
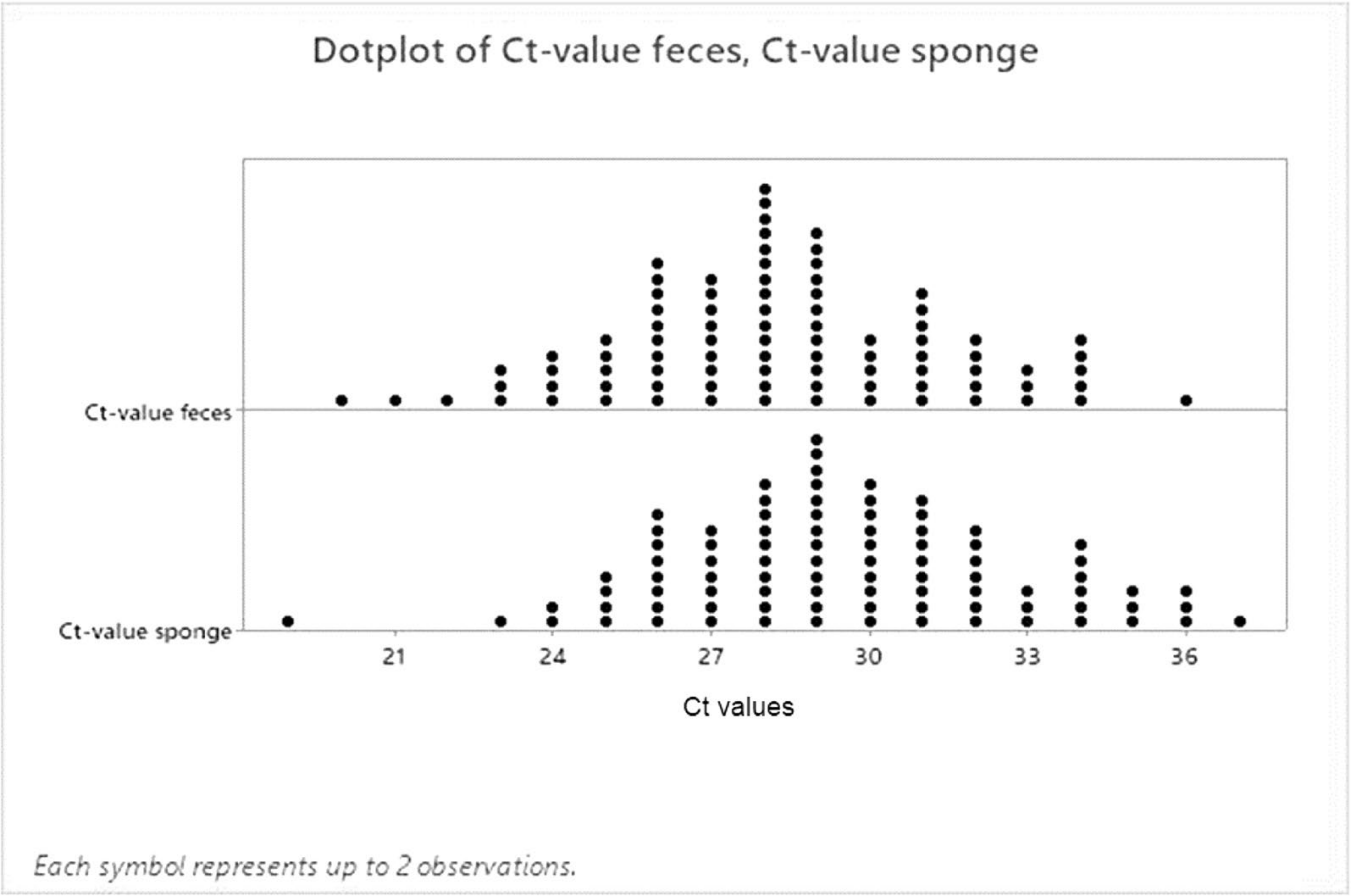
Distribution of Ct values obtained via different sampling methods

Figure 1 shows the distribution of the results of the qPCR and the respective CT values. Faecal samples presented slightly greater DNA masses (1 CT value difference expressed as the median) than did samples collected from sponges.

**Fig. 2:**
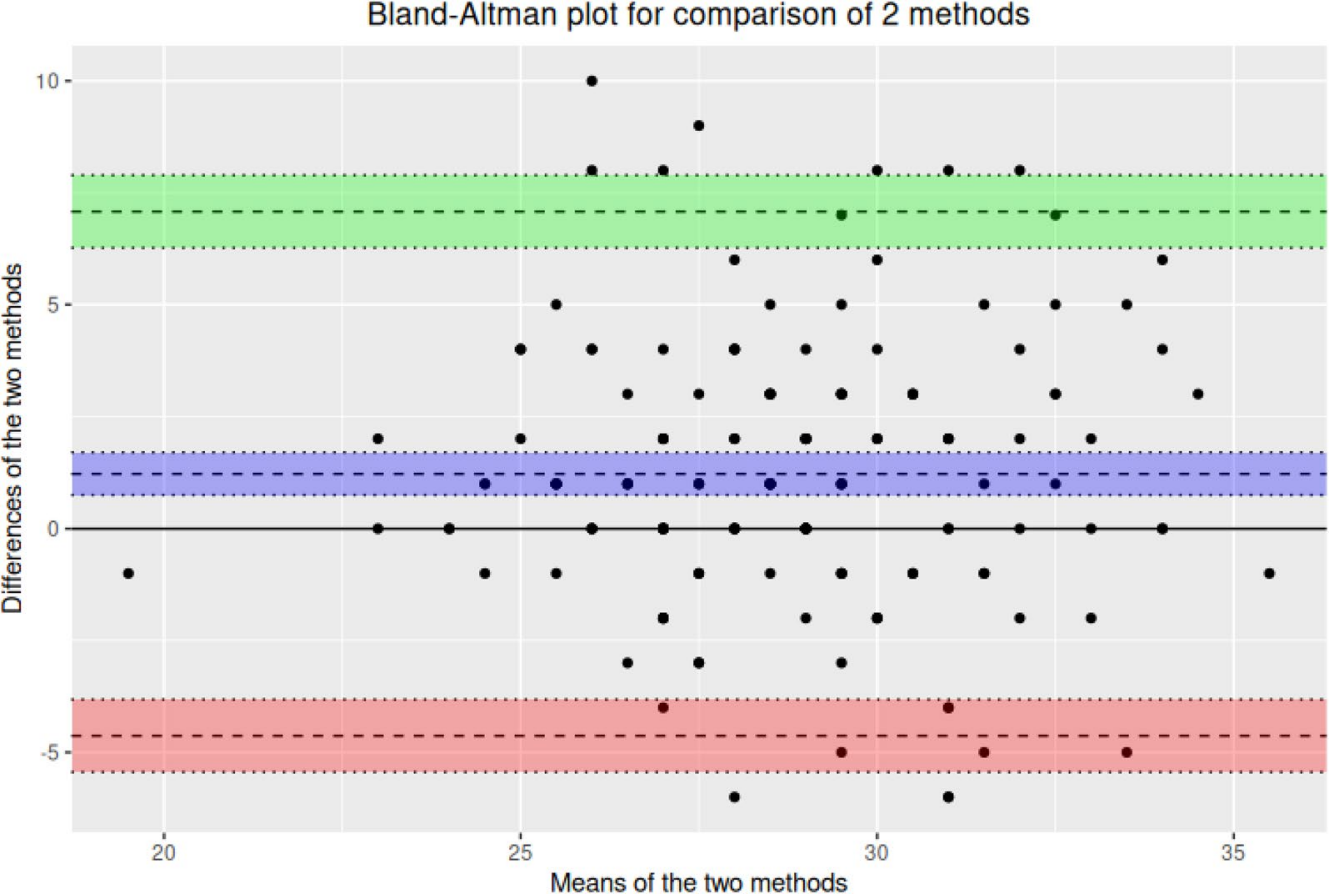
The Bland‒Altman analysis of the two sampling methods revealed a significant bias in the Ct values between the samples (*p* < 0.001)

A correlation coefficient of r = 0.51 was calculated, revealing moderate positive relationships between both sampling methods. The Bland‒Altman test revealed a significant bias in the CT values between the samples (*p* < 0.001), with an average difference of 1.22 95% CI [0.75–1.70] (Fig. 2).

The specific evaluation of the influence of the consistency of faecal samples (FSs) on the CT value tendency towards a higher DNA load with samples with pasty and semiliquid consistency, whereas in the case of the sponge technique, the difference was significant, except for the comparison of FS 2 vs. FS 3 (*p* = 0.196), where only a numerical difference was observed. Only numerical differences were observed for faecal samples: (*p* = 0.741; *p* = 0.473; *p* = 0.704 for FS1 vs. FS2, FS1 vs. FS3 and FS2 vs. FS3) (Table 2).

**Table 2:** Effect of the consistency of faecal material collected via sponges on the CT value

	Faecal score: sponges		
	1	2	3
N	61	169	20
Mean ± SD (CT)	31.04 ± 3.80	29.48 ± 2.98	28.12 ± 2.09
Median	31.00	29.00	28.00
[Q1–Q3]	[30.00–34.00]	[27.00–31.00]	[27.00–30.00]
[Min–Max]	[19.00–36.00]	[23.00–37.00]	[24.00–31.00]
	Faecal score: faecal samples		
N	61	169	20
Mean ± SD (CT)	28.76 ± 4.05	28.29 ± 2.98	27.62 ± 1.96
Median	29.00	28.00	28.00
[Q1–Q3]	[26.00–32.00]	[26.00–30.00]	[26.00–28.00]
[Min–Max]	[20.00–36.00]	[22.00–36.00]	[24.00–31.00]

SD = standard deviation, Q1 = first quartile, Q3 = third quartile, Min = minimum, Max = maximum, CT value = threshold cycle

## Discussion

In the present study, we compared two sampling approaches to detect the DNA of *C. suis* via species-specific qPCR. The standard collection of faecal samples and the use of sponges for environmental sampling were compared, with the objective of developing an alternative and more convenient method of sample collection for the detection of *C. suis*. Previous studies comparing different sampling methods, mainly in the field of bacteriology, have shown that environmental sampling methods are more efficient than testing methods based on sampling individuals. Compared with the sampling of individual piglets, the sponge technique is considered a less invasive approach, reducing the amount of labour and time needed by approximately 50% (personal observation) [21, 22, 26]. Similar assessments for parasitic infection of piglets are lacking, mainly due to the substantial differences in the requirements for the diagnosis of different bacterial pathogens in terms of sample collection for cultivation. The characteristics of *C. suis* and its ability to survive in the environment of a farm are favourable for the evaluation of different environmental sampling techniques [27]. Sponges can also be used for the verification of cleaning and disinfection protocols for pig pens; currently, there is not a standardized and reproducible method to measure the infectious load (contamination of farrowing crate) and to monitor the impact of cleaning and disinfection on parasite load.

Monitoring diseases, including cystoisosporosis, in populations of piglets by collecting individual faecal samples represents a major limitation because of the large number of piglet samples that are required to detect infection, making the process expensive and time-consuming. In addition to the limitations mentioned above, field diagnostics of cystoisosporosis are considered particularly challenging tasks by field veterinarians because of the specific features of *C. suis* infection in piglets [8, 9]. Piglets within one litter and in different litters in a farrowing barn are not infected at the same time; individual animals show biphasic excretion with a relatively short duration of oocyst excretion (usually 5 days) [1, 28]. Similarly, the duration of diarrhoea is rather short and is not necessarily associated with the presence of oocysts or the shedding of oocysts into the environment. For these reasons, the standard samples—faecal samples from piglets—are obtained by repeated sampling (2nd and 3rd weeks of age) targeting pools from at least 5 piglets/litter [8, 9, 29].

Previous studies confirmed that the detection of the presence of parasite DNA (PCR may detect small amounts of parasite DNA in cells shed with the faeces in the pre- or postpatent periods), with molecular methods showing a higher sensitivity compared with conventional parasitological methods [20]. The use of various diagnostic methods based on PCR protocols simplifies the sampling procedure and logistics of shipment to the laboratory, as does the use of both fresh and as frozen samples [11, 20]. The disadvantage of the PCR method is that it cannot detect oocyst shedding exclusively; instead, other intracellular stages are also detected. For that reason, its suitability for the evaluation of treatment protocols is limited unless the investigation is not accompanied by visualization of oocysts via other methods. It was previously speculated that toltrazuril may inhibit the formation of oocysts but not the infection and early development of the parasite, thus leading to the excretion of intracellular stages, which can be detected via PCR [20].

Our results indicate that the collection rates of faecal samples at the litter or farm level are similar to those of environmental samples (i.e., surfaces of farrowing crates). According to our findings, the frequency of positive detection of parasite DNA by qPCR did not differ at the farm level, where 100% of the farms were positive and correctly identified by both types of sampling, taking into consideration paired samples representing individual litters. The difference in positivity status was observed in only 10.8% of the paired samples. The time needed for collection was reasonably shorter when sponges and the suggested protocol were used (10 min vs. 15 min per farm investigated; personal observation).

Both the sensitivity and specificity of sponge sampling and the collection of faecal samples, the latter of which is the gold standard procedure, were high. Although the sponge method was not evaluated with a perfect reference standard, comparison with standard faecal sampling shows that the sponge method provides a suitable alternative to standard sample collection, with a sensitivity of 91.2%. A specificity of 84% is generally considered good, though not excellent, and this result needs to be considered in the clinical context, for example, for initial screening combined with other diagnostic tools (e.g., AF) to evaluate control measures (e.g., implementation of therapeutic programs) to reduce the risk of misdiagnosis. The limitation of our pilot study is that other standard diagnostic techniques (AF and flotation) were not used in parallel, which could be a feature in future studies.

From that perspective, the use of sponges for collecting organic material from the farrowing crate environment can be considered a good alternative to the more laborious and time-consuming collection of faecal samples, which was considered the gold standard sampling technique in our study.

Further assessment, which may improve the understanding of various sampling protocols, would need to use defined samples (spiked with oocysts) with different water contents in the faecal matrix (FS); however, the limitations of such approaches would need to be considered, as it is typical for the faecal samples of piglets to have different fat contents in the first weeks of life.

Compared with the load of parasite DNA, the qPCR-positive CT values were lower for the sponges, and the faecal samples presented significantly higher DNA masses, with an average difference of 1.22 (95% CI [0.75–1.70]). Future studies should evaluate such differences in greater detail and explore differences in the exact number of copies of DNA. This difference might be caused by the lower typical amount of faecal matter in samples obtained from sponges. The effect of the faecal score (FS) (consistency and water content) can also influence the results of qPCR. Semiliquid and pasty samples presented higher concentrations of parasite DNA than did samples from solid faeces for both sampling techniques, with a significant effect on sponges. This result is consistent with the general protocol of microbiology for environmental sampling, in which moisture and a relatively high content of water from either surfaces or sampling devices are required for effective sampling of surfaces [30, 31]. To support this observation, more samples should be investigated, and FS4-type faeces, which represent liquid diarrhoea, need to be investigated. The practical limitation of collection with sponges is the difficulty of evaluating the FS, which depends on the amount of faecal matrix collected via this approach. Previously, mainly pasty faecal samples were associated with the presence of oocysts and provided a greater chance to detect *C. suis* in pooled samples [9]. One of the limitations of the use of sponges is the rather difficult homogenization of material collected from the surface of the farrowing crate, which may result in a lower amount of DNA obtained from samples. This disadvantage might be compensated for by the ease of screening and the possibility of increasing the number of litters included.

Our findings suggest that environmental sampling strategies could be considered for *C. suis* in farm diagnostic and surveillance programs, particularly if the goal is merely to detect the presence of parasites and identify farms at risk. A limitation of our study is that only farms with a history of *C. suis* infection were included; nevertheless, seven farms (7/25) later implemented a toltrazuril-based program, which involved the use of conventional methods for the detection of oocysts and which may have led to these farms controlling or even eliminating *C. suis* infection. To gain a more precise understanding of the potential of sponge-based techniques, the status of farms should be evaluated before the start of future studies, and other available techniques (e.g., (flotation, AF) should be used in parallel. This study provides new information on sampling approaches to conduct effective cystoisosporosis surveillance in piglets and identifies sponges as a novel sample type that offers a convenient, inexpensive and sensitive way to monitor this frequent parasitic infection in piglets prior to weaning. In addition to specific treatment, optimized hygiene, including chemical disinfection, is considered a similar essential part of the control of cystoisosporosis together with metaphylactic treatment [32]. The sponge sampling method would be a good alternative and suitable control tool for biosecurity measures applied on farms (cleaning and disinfection). This aspect needs to be further investigated after the results of this pilot study. Moreover, PCR-based protocols should not replace diagnostic methods for *C. suis* infection in piglets, which are based on the detection of the final stage of parasites in faecal samples (oocyst shedding), and they should not replace evaluation with metaphylactic programs or the detection of possible resistance issues.

A limitation of our study is that all farms were known to be *C. suis* positive, so farms that were previously negative or without screening for *C. suis* must be sampled in the future to confirm the usefulness of the assay.

## Conclusions

Our findings suggest that environmental sampling strategies could be considered for *C. suis* on-farm diagnostic and surveillance programs with a high level of sensitivity and acceptable specificity compared with standard collection methods based on faecal samples. This study provides new information on sampling approaches to conduct effective cystoisosporosis surveillance in piglets. The use of sponges in combination with qPCR may offer a convenient and sensitive method to monitor this frequent parasitic infection in piglets prior to weaning. For screening, we suggest a minimum sample size of 10 litters or 10% of a farm’s litters (on large farms). The limitation of this pilot study results is that the first evaluation was performed on farms historically positive for *C. suis*. For future studies, evaluation of the suitability of the technique should be performed on the basis of comparisons with methods for visualizing oocysts (flotation and AF), together with the establishment of detection limits.

## Supplementary Information

Additional file1 (DOCX 26 kb)

Additional file2 (DOCX 33 kb)

Additional file3 (DOCX 60 kb)

## Data Availability

All the data are included in this publication.
