# Supplementary material for: Assessment of sponge sampling for real-time PCR detection of Cystoisospora suis from environmental and faecal samples from piglet-producing farms
Source: Porcine Health Manag. 2025 Jul 31;11:43. doi: 10.1186/s40813-025-00454-5 (PMC12315398; doi:10.1186/s40813-025-00454-5)
Supplement: Supplementary file 1 — Additional file1 (DOCX 26 kb) [file 40813_2025_454_MOESM1_ESM.docx]

**Consent of the animal owner/farm or the person responsible for the animals**

I, the undersigned,

declare myself on behalf of my company

Company _____________________________________________________________________________

Adress____________________________________________________________________________

I agree that my animals (or animals under my responsibility) participate in the studies *"Evaluation of the prevalence of Cystoisospora suis in German piglet farms using a novel real-time PCR"* and *"Validation of a new sampling method for a real-time PCR for the detection of Cystoisospora suis from environmental and fecal samples"*. The studies are sponsored by Ceva Tiergesundheit GmbH (Kanzlerstraße 4, 40472 Düsseldorf, Germany) and Ceva Santé Animale (10 Avenue de la Ballastière, 33500 Libourne, France) and carried out by VT Services GmbH (Carl-Benz Straße 21, 48734 Reken, Germany) and Bio-Diagnostix Labor GmbH (Carl-Benz Straße 21, 48734 Reken, Germany).

The aim of the studies is (i) to determine how prevalent the pathogen *Cystoisospora suis* is on piglet-producing farms in Germany and (ii) to validate a new sampling method for *Cystoisospora suis* real-time PCR.

I was informed by the auditor:

- about the aim of the studies.
- on the conditions for the evaluation of the animals.
- about my right to remove animals from the studies.

Accordingly, I undertake the following:

- present my animals at each visit scheduled in the study protocol.
- to follow the instructions I have received for the proper conduct of the studies.
- to provide these animals with adequate care throughout the study period.
- report to the inspector any changes in the health status of the animals and any significant events that occurred during the inspection period.
- to maintain confidentiality with regard to these studies and not to disclose any information about them.

Only operational data is processed for the studies. Personal data is not processed. The study results are published anonymously.

Signed in duplicate in (place) _________________, at __________________

Signature of the owner Signature of the investigator:

(or the operational manager)

_____________________________________ ____________________________________
