# Supplementary material for: Assessment of sponge sampling for real-time PCR detection of Cystoisospora suis from environmental and faecal samples from piglet-producing farms
Source: Porcine Health Manag. 2025 Jul 31;11:43. doi: 10.1186/s40813-025-00454-5 (PMC12315398; doi:10.1186/s40813-025-00454-5)
Supplement: Supplementary file 2 — Additional file2 (DOCX 33 kb) [file 40813_2025_454_MOESM2_ESM.docx]

**Quick guide for investigator**

1. The participating farms are approached as part of routine visits, preferably no later than Wednesday.
2. All investigators receive a study kit with all the necessary information/materials from the sponsor.
3. The farmer consented to participate in this study and signed Supplementary material 1.
4. In the participating farm, 10 farrowing pens are selected for the actual sampling.
   1. Sampling age as well as fecal consistency are chosen in order to detect as many positive samples as possible, which is known for each farm from previous samplings.
   2. Inspector 1 collects per farrowing pen
      1. Five individual fecal samples from the floor into a collection tube. Ideally, the sampling tube should be 75% full and no less than "to the lower ring".
      2. A single wipe sample with the enclosed sponges, first wiping the (i) accessible walls of the farrowing crate and then the (ii) floor.
5. Supplementary material 3is filled out in accordance with the fecal tubes and sponges. 5 tubes (1a-1e / 2a-2e) are placed in each plastic bag, the sponges are packed individually in bags. The study sheet is placed in the study kit together with the plastic bags, sealed and quickly taken to the practice and stored refrigerated.
6. The study kit is best collected on the same day by a logistics company and brought to the laboratory.
