# Supplementary material for: Assessment of sponge sampling for real-time PCR detection of Cystoisospora suis from environmental and faecal samples from piglet-producing farms
Source: Porcine Health Manag. 2025 Jul 31;11:43. doi: 10.1186/s40813-025-00454-5 (PMC12315398; doi:10.1186/s40813-025-00454-5)
Supplement: Supplementary file 3 — Additional file3 (DOCX 60 kb) [file 40813_2025_454_MOESM3_ESM.docx]

**Validation study / data sheet**


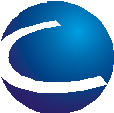


Farm name: ______________________________________________________________________

Date: _____________________________ Reviewer ID:______________________________

| **Sample number collection tube** | **Sample number**  **Sponge** | **Fecal score** | **Sow identification/ parity** | **Farrowing date** |
| --- | --- | --- | --- | --- |
| **1a** | **1** |  |  |  |
| **1b** | 2 |  |  |  |
| **1c** | 3 |  |  |  |
| **1d** | 4 |  |  |  |
| **1e** | 5 |  |  |  |
| **2a** | 6 |  |  |  |
| **2b** | 7 |  |  |  |
| **2c** | 8 |  |  |  |
| **2d** | 9 |  |  |  |
| **2e** | 10 |  |  |  |
